# Genomic characterization of ceftazidime/avibactam-resistant *Klebsiella pneumoniae*: a retrospective analysis on clinical isolates from an Italian transplant unit

**DOI:** 10.3389/fmicb.2026.1694693

**Published:** 2026-04-02

**Authors:** Emanuele Nicitra, Maddalena Calvo, Ilenia Martina Pia Filannino, Nicolò Musso, Grete Francesca Privitera, Davide Lo Porto, Andrea Cona, Francesco Monaco, Alessandra Mularoni, Stefania Stefani, Dafne Bongiorno

**Affiliations:** 1 Section of Microbiology, Department of Biomedical and Biotechnological Science, University of Catania, Catania, Italy; 2 U.O.C. Laboratory Analysis Unit, University Hospital Policlinico-San Marco, University of Catania, Catania, Italy; 3 Faculty of Medicine and Surgery, "Kore" University of Enna, Enna, Italy; 4 Unit of Bioinformatics, Department of Clinical and Experimental Medicine, University of Catania, Catania, Italy; 5 Unit of Infectious Diseases, ISMETT-IRCCS Istituto Mediterraneo per i Trapianti e Terapie ad Alta Specializzazione, Palermo, Italy

**Keywords:** antimicrobial resistance, ceftazidime/avibactam, *Klebsiella pneumoniae*, molecular characterization, resistome, transplant unit, virulome

## Abstract

**Introduction:**

*Klebsiella pneumoniae* infections are a significant healthcare concern due to advancing antimicrobial resistance rates and virulence genes’ detection. Specifically, ceftazidime/avibactam resistance is increasingly diffused among *K. pneumoniae* clinical isolates, limiting therapeutic choices and complicating patients’ outcomes. Some authors documented the simultaneous presence of ceftazidime/avibactam resistance markers and specific virulence genes. According to these premises, we characterized ceftazidime/avibactam-resistant *K. pneumoniae* strains, exploring their diffusion at the Mediterranean Institute for Transplants and Highly Specialized Therapies (ISMETT) of Palermo, Italy.

**Materials and methods:**

A total of 14 ceftazidime/avibactam-resistant strains were retrospectively analyzed using whole-genome sequencing and Sanger validation.

**Results:**

Most strains belonged to ST101 and carried *bla_KPC-31_, bla_KPC-3_*, or *bla_KPC-34_* genes, often with resistance-associated mutations. The analysed strains revealed multidrug resistance, frequent outer membrane protein alterations, and a significant spectrum of virulence genes. Additionally, plasmid analysis revealed sequence type–specific replicons potentially involved in gene dissemination.

**Discussion:**

These findings highlight a convergence of resistance and virulence traits in high-risk *K. pneumoniae* clones, enhancing the importance of MDR pathogens’ genomic surveillance in critical healthcare settings.

## 1 Introduction

Antimicrobial resistance reached a concerning worldwide incidence during the last decades, specifically referring to *Klebsiella pneumoniae* as one of the most diffused multidrug-resistant bacteria. This Gram-negative pathogen often reports resistance to the beta-lactam group, mainly depending on carbapenemase production. Furthermore, decreased cell permeability, loss of outer membrane proteins (Omps), and efflux pumps may contribute to beta-lactam resistance episodes (Karampatakis et al., 2023; Carattoli et al., 2021).

On the premise of *K. pneumoniae’s* remarkable colonization and dissemination within the human host, carbapenemase-producing strains became a major threat within healthcare settings, complicating therapeutic choices and critical patient management (Karampatakis et al., 2023; Carattoli et al., 2021). Literature data documented eight major multi-drug resistant (MDR) *K. pneumoniae* clonal groups (CG), whose denominations are CG15, CG20, CG29, CG37, CG147, CG101, CG258, and CG307. Remarkably, CG307, CG101, CG258, and CG147 emerged as common healthcare-associated infection etiological agents (Carattoli et al., 2021).

Novel beta-lactam/beta-lactamase inhibitor combinations (BLICs) contrasted the MDR for *K. pneumoniae* infections within critical hospital settings during the last decade. Ceftazidime/avibactam (CZA) combines a broad-spectrum third-generation cephalosporin (ceftazidime) and a beta-lactamase inhibitor (avibactam). This combination showed promising effectiveness due to the avibactam active action against Ambler classes A, C, and D beta-lactamases, along with numerous extended-spectrum beta-lactamases (ESBL), *K. pneumoniae* carbapenemases (KPC), oxacillinases (OXA-48), and cephalosporinases (AmpC) (van Duin and Bonomo, 2016; Stachyra et al., 2010; Aktaş et al., 2012). Several data demonstrated ceftazidime/avibactam reduced susceptibility or resistance among *K. pneumoniae* isolates, mainly due to metallo-beta-lactamase (MBL) production. Additionally, beta-lactamase variants, membrane permeability alterations, penicillin-binding proteins (PBPs), and efflux pumps contribute to the same phenomenon (Campogiani et al., 2023). Regarding KPC-producing *K. pneumoniae*, previously published studies reported *Ω*-loop mutations (insertion, substitutions, or deletion) in the blaKPC gene, such as the D179Y deletion (Alsenani et al., 2022). The ceftazidime/avibactam-resistant *K. pneumoniae* isolates further reduced therapeutic alternatives in the case of severe infections. Moreover, these MDR strains showed a consistent dissemination capability, inspiring hypotheses about the simultaneous virulence factors (Xiong et al., 2022; Antinori et al., 2020; Livermore et al., 2015; Winkler et al., 2015; Cuzon et al., 2011). These considerations required specific investigation and infection control measures to contain these clones’ diffusion within critical healthcare settings.

According to these assumptions, we propose an experimental analysis of ceftazidime/avibactam-resistant *K. pneumoniae* strains belonging to immunocompromised patients. The study aimed to investigate ceftazidime/avibactam resistance molecular mechanisms and eventual *K. pneumoniae* virulence factors through next-generation sequencing technologies.

## 2 Materials and methods

### 2.1 Strains sampling

A retrospective study included ceftazidime/avibactam-resistant *K. pneumoniae* strains from the Mediterranean Institute for Transplantation and Highly Specialized Therapies (ISMETT) in Palermo, Italy. This Institute includes high-specialization transplant units for abdominal and cardiothoracic surgeries, along with their corresponding intensive care units. We analyzed single isolates from fourteen different patients recovered during a 1-year and 10-month period (February 2021–December 2022). The analysis specifically regarded ceftazidime/avibactam-resistant *K. pneumoniae* strains isolated from infection episodes, excluding colonizing isolates. Specifically, deep respiratory, blood, and urine *K. pneumoniae* isolates were included as infection specimens. Otherwise, *K. pneumoniae* emerging from surveillance samples (e.g., rectal or oropharyngeal swabs) were excluded, only indicating a bacterial colonization.

Identification and antimicrobial susceptibility had previously been performed by the Vitek 2 system (bioMérieux, Marcy l’Etoile, France) at the above-mentioned hospital. These strains were forwarded to the Molecular Microbiology and Antimicrobial Resistance (MMAR) Laboratory at the University of Catania (Catania, Italy), aiming to investigate *K. pneumoniae* resistome and virulome patterns. Clinical data and microbiological reports from all the involved patients were collected into a datasheet according to the Ethical Committee’s favourable opinion.

### 2.2 Phenotypic assays and antimicrobial susceptibility testing

The phenotypic assay known as the “string test” was performed on the included isolates. Despite the subsequent availability of whole-genome sequencing, the string test was used as an initial phenotypic, low-cost, and immediate screening tool to assess hypermucoviscosity, enabling the integration of genomic data with functional phenotypic information. The assay tested positive in the case of a thread-like string longer than 5 millimeters for a stretched *K. pneumoniae* colony through a sterile loop from the agar plate. The authors aimed to register this result for a subsequent comparison to the virulome patterns details. The EUCAST broth microdilution (https://www.eucast.org/fileadmin/src/media/PDFs/EUCAST_files/MIC_testing/Reading_guide_BMD_v_5.0_2024.pdf) confirmed all the *K. pneumoniae* strains’ susceptibility profiles, reporting definitive minimum inhibitory concentrations (MICs) values for ceftazidime/avibactam (CZA), amoxicillin/clavulanate (AMC), piperacillin/tazobactam (TZP), cefepime (FEP), ceftazidime (CAZ), meropenem (MEM), imipenem (IMI), aztreonam (AZT), meropenem/vaborbactam (MEV), ciprofloxacin (CIP), amikacin (AK), gentamycin (CN), and trimethoprim/sulfamethoxazole (SXT). All the gathered MIC values followed the EUCAST guidelines interpretation criteria (EUCAST, 2025).

### 2.3 Whole-genome sequencing

All the collected strains underwent a DNA extraction procedure through QIAGEN QIAamp® DNA Mini Kit (Ref. 51,304, QIAGEN, 40724 Hilden, Germany), according to the manufacturer’s instructions. Subsequent DNA quantification used the Eppendorf BioPhotometer® D30 and the fluorimeter Qubit dsDNA BR Assay Kit. These tools allowed a purity and quantity evaluation of the initial sample (Ref. 32,850, Invitrogen, 92,008 Carlsbad, CA, USA) (Bongiorno et al., 2022). The Illumina MiSeq platform managed the next-generation sequencing (NGS) analysis using 100 ng of each extracted sample.

This procedure followed the manufacturer’s instructions provided by the Watchmaker’s DNA Library Prep kit with Fragmentation–Watchmaker Genomics® (Ref. 7 K0013-024, 5,744 Central Avenue, Suite 100, Boulder, CO 80301, USA). Twist Universal Adapter System provided indexes (16 Indexes, 16 Samples) (Ref. 101,307, Twist Bioscience, HQ 681 Gateway Blvd, South San Francisco, CA 94080FAQ). The fluorometric Qubit dsDNA HS Assay Kit (Ref. Q32851, Invitrogen, Carlsbad, CA 92008, USA) quantified libraries, and the Agilent® High Sensitivity DNA Kit (Ref. 5,067–4,626) evaluated their quality. Illumina® provided the “Denature and Dilute Libraries Guide” to denature and dilute libraries, choosing 8.5 pM as the loading concentration. Finally, sequencing analysis utilized MiSeq Reagent Kits v3 (Ref.15043895, Illumina, Inc., 92,122, San Diego, CA, USA). The Local Run Manager v3 software created the Sample Sheet according to the corresponding software guide by Illumina (Calvo et al., 2024; Nicitra et al., 2025).

### 2.4 Sanger sequencing

In order to confirm mutations potentially involved in resistance to ceftazidime/avibactam and to achieve optimal coverage and very high base-level accuracy in the specific region assessed for the presence of a point mutation, all KPC-3 strains underwent Sanger sequencing of this gene. Primers were designed to cover the gene region responsible for resistance (primer sequences available in Supplementary Table 2). Polymerase chain reaction (PCR) was performed on strains harboring the *bla*_KPC-3_ gene (isolates 1, 4, 11, 13, and 14) using the QIAGEN Multiplex PCR Kit (Ref. 206,143, QIAGEN, 40724 Hilden, Germany). PCR products were then purified using the ExoSAP-IT™ PCR Product Cleanup Reagent (Ref. 78,205, Applied Biosystems™, Thermo Fisher Scientific Inc., 168 Third Avenue, Waltham, MA 02451, USA) before sequencing and labelling with the BigDye™ Terminator v3.1 Cycle Sequencing Kit (Applied Biosystems™, Thermo Fisher Scientific Inc., 168 Third Avenue, Waltham, MA 02451, USA). Further purification was carried out using the BigDye XTerminator™ Purification Kit (Applied Biosystems™, Thermo Fisher Scientific Inc.). Prior to sequencing, all purified products were denatured at 93 °C for 3 min using 10 μL of formamide. The final products were then sequenced using the Applied Biosystems™ SeqStudio™ Genetic Analyzer (Applied Biosystems™, Thermo Fisher Scientific Inc.). Sequences were subsequently analyzed and compared with the KPC-3 wild-type gene (ARO:3002313) using FinchTV (Chromatogram Viewer v1.5).

### 2.5 Data analysis

The QIAGEN CLC Genomics Workbench software analyzed the obtained data following the User Manual for the CLC Microbial Genomics Module v22.0 released on 4 January 2022 (QIAGEN, Aarhus, 8,000 Denmark). This software used the CARD database (https://card.mcmaster.ca/) to define resistance, virulence, and sequence typing (MLST) genes. Fourteen paired-end bacterial raw reads were trimmed with TrimGalore (v0.5.0) (Martin, 2011; Krueger, 2025) to remove the adapter sequence. Furthermore, the bacterial genome was *de novo* assembled using Unicycler (v0.4.8) (Wick et al., 2017) with the Illumina-only assembly modality. Virulence factors, resistance genes, and capsule loci are derived from Kleborate (v2.2.0) and the Kaptive command (Lam et al., 2021; Wyres et al., 2016). The software Prokka (v1.13) defined bacterial annotation. Moreover, Unicycler output assemblies were aligned with several protein sequences to identify punctual mutations (Seemann, 2014). Heatmaps emerged from the R package Complex Heatmap software (Danecek et al., 2021). Plasmid analysis used the software Bactopia (https://pubmed.ncbi.nlm.nih.gov/32753501/) and its “bactopia tools” PlasmidFinder (Li, 2011).

The software Tormes confirmed virulence and resistance genes (Sievers et al., 2011). The samples were screened for the *mag*A gene using AC: AY762939. ClustalO (Price et al., 2010) allowed phylogenetic tree creation, reporting alignment of sequences, and FastTree (Paradis and Schliep, 2019). Additionally, the tree illustration was developed from the R packages showing *ape* (Yu et al., 2017), *ggtree* (RColorBrewer, 2025), RColorBrewer (ggplot2, 2025), *ggplot2*, and *ggn* new scale (Campitelli, 2019). The reports of the quality score for the sample are available in Supplementary Table 3.

## 3 Results

### 3.1 Ceftazidime/avibactam exposure

The 14 analyzed strains belonged to immunocompromised patients from an Italian Transplant Unit, during 1 year (2021–2022). Seven patients (50%) recorded previous CZA exposure, while six isolates (42.8%) reported carbapenem regimens. Microbiological investigations isolated CZA-resistant *K. pneumoniae* within 60 days after CAZ or carbapenem therapeutic approaches. Four patients (28.6%) revealed a KPC-producing *K. pneumoniae* colonization before the infection episode. Among these cases, strains 4, 5, 10, and 12 emerged from patients documenting previous stays in other hospital settings before the ISMETT admission. Regarding strains 5 and 12, the patient’s clinical history is registered as having Nigerian and Greek origins. The clinical reports recorded death for five patients (35.7%) due to CZA-resistant *K. pneumoniae* severe infection. Patients’ clinical features and microbiological details are reported in table 1 and Supplementary Table 1.

### 3.2 Sample features

The study analyzed 14 clinical isolates demonstrating CZA resistance and infection aetiology. The susceptibility profile confirmed resistance to amoxicillin/clavulanate, piperacillin/tazobactam, cefepime, ceftazidime, ciprofloxacin, and gentamycin for all the strains (100%). Otherwise, 13 isolates (92.8%) reported aztreonam and amikacin resistance. Ten *K. pneumoniae* (71.4%) showed trimethoprim/sulfamethoxazole resistance. Finally, resistance to meropenem, imipenem, meropenem/vaborbactam, and colistin emerged in two isolates (14.2%).

The NGS analysis revealed three different sequence typings. Specifically, this procedure confirmed ST101 for Italian patients (85.7%), while ST39 and ST307 (cases 5 and 12) emerged for 1 strain each (7.1%), respectively from Nigeria and Greece. All the isolates revealed a similar genotypic profile, reporting LPS O-antigen D-galactans encoded by the O1/O2v1 serotype in ST101 or O1/O2v2 serotype in ST39 and ST307. These serotypes differed by rearrangements in the rfb region. The analysis registered different capsular polysaccharides (k-locus) as K17, K23, and K102, respectively related to the wzi gene sequences 137, 172, and 137. Supplementary Table 1 summarizes the year, source, susceptibility profile, and genomic sample features.

### 3.3 Resistome analysis

**Figure 1:**
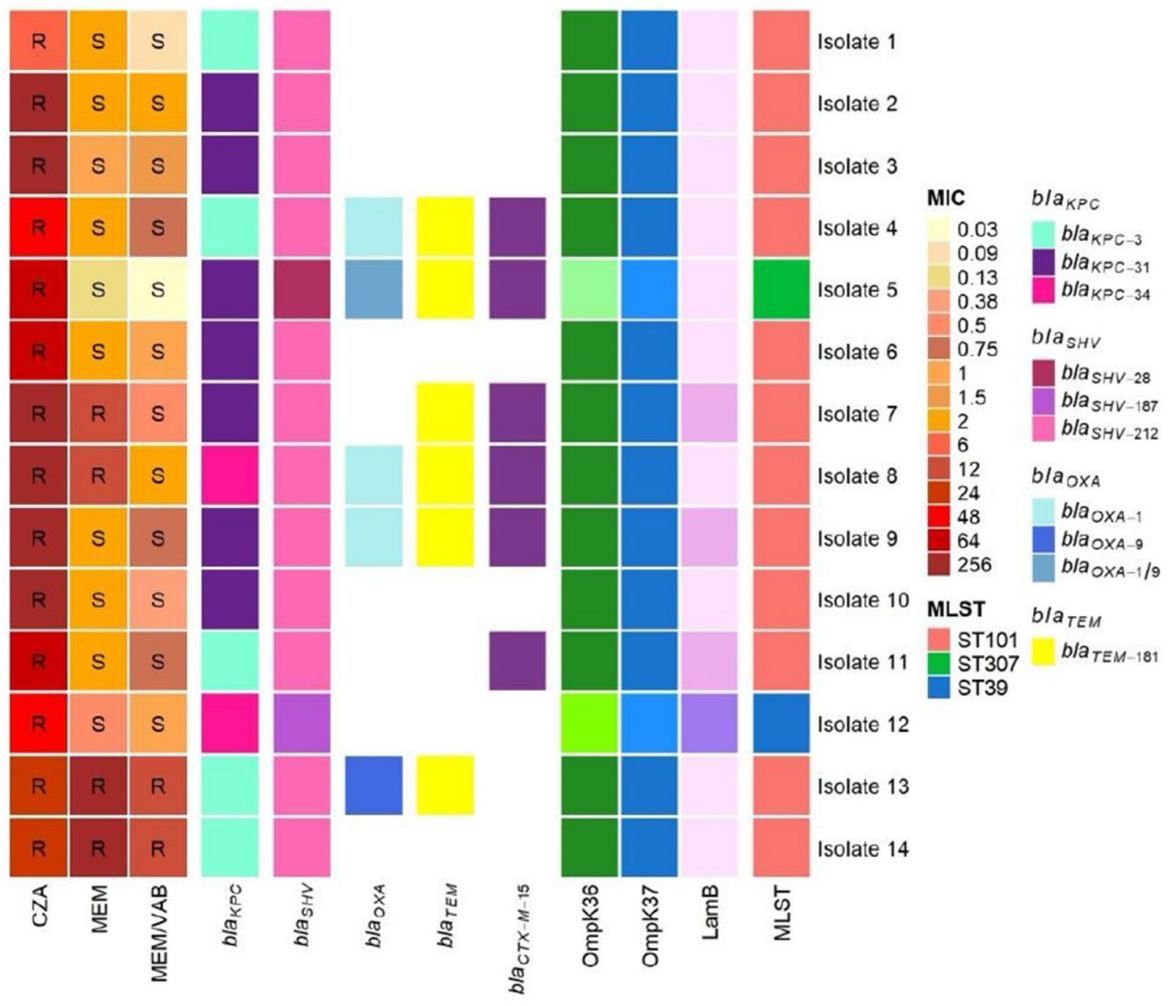
Resistome details. The image reports antibiotic susceptibility (S) or resistance (R), detected beta-lactams’ resistance genes, and *omp* genes mutations for all the collected strains. OmpK36 dark green indicates the following mutations: A193G-Y201F-Y210W-N221H-L225T-G226D-206 D227E-K231V-L232P-T258S-E308D-H349R; case 5 mutations regard the following positions: A183T-T192G-Y201F-N221H-L225N-N229K-207$2301-S271F-L3071-1315 L-D344E-S346DH349R-N350R; case 12 instead is mutated only in two positions (V178P-H349R). OmpK37 in case 5 and case 12 are mutated in: N26169M–1,127 M-N229G-Q234H-E243D- D274G-V2761; the other cases are mutated in: N26-169 M-1127 M. Finally, LamB in case 12 is mutated in K49Q-L75V-N94Q-D97N-S103N-A109V–1,111 V; case 7, case 9, and case 11 possess a stop in position Q112,” other samples do not show mutations. S, susceptible; R, resistant, CZA, ceftazidime/avibactam; MEM, meropenem; MEM/VAB, meropenem/vaborbactam; MLST, multi-locus sequence typing: MIC, minimum inhibitory concentration.

We compared phenotypic susceptibility profiles to genotypic details derived from the sequencing analysis. Figure 1 includes antibiotic susceptibility (S) or resistance (R), detected beta-lactam resistance genes, and the eventual outer membrane proteins (Omps) gene mutations. As regards the blaKPC gene, several variants emerged: five strains (35.7%) documented blaKPC-3, seven strains (50%) showed blaKPC-31, which is directly involved in CZA resistance episodes; two isolated 14.3% revealed blaKPC-34 with D179Y and H281Y associated mutations.

**Figure 2:**
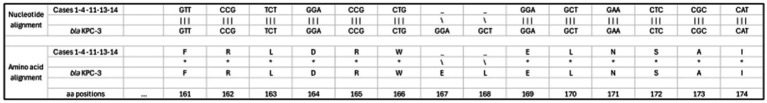
Partial alignments of nucleotide and corresponding amino acid sequences of the *blaKPC-3* gene from clinical *Klebsiella pneumoniae* isolates, illustrating a six-nucleotide deletion within the Q-loop region. The affected residues may contribute to reduced susceptibility to ceftazidime-avibactam.

Strains harboring blaKPC-3 recorded a CZA MIC range of 12–24 mg/L, while the highest values (MIC range: 64- > 256 mg/L) were developed from blaBPC-31 and blaKPC-34 isolates. Regarding blaKPC-3, 60% of the detected genes presented a two-amino acid deletion (Glu and Leu) at 167–168 positions. Isolates 1, 4, 11, 13, and 14 documented this condition, further analyzed and confirmed through Sanger sequencing (Figure 2). Furthermore, all strains showed blaSHV genes as blaSHV-212 (85.7%), blaSHV-28 (case 5), and blaSHV-187 (case 12). Three strains revealed blaOXA-1 and blaTEM-181, while one showed blaOXA-9 and blaTEM-181, and another showed blaOXA-1/9 and blaTEM-181.

On the other hand, one strain reported only blaTEM-181 without any blaOXA genes. Six isolates (42.8%) carried blaCTX-M-15. As regards membrane permeability alterations, all the strains outlined a wild-type *ompK35*. Moreover, all ST101 isolates shared the same mutation set in the *ompK36* and *ompK37* genes. All ST101 strains share the same mutation set in the *ompK36* and *ompK37* genes. An identical mutation pattern in the *ompK36* and *ompK37* genes originated from ST307 and ST39. Isolates 7, 9, and 11 revealed a synonymous mutation in *lamB*, while case 12 showed a significant *lamB* mutation pattern. A core resistance gene group appeared in all the collected isolates, reporting oqxA-B for efflux pump against quinolones, while five strains (35.7%) qnrB17 encoding peptides for alternative fluoroquinolones targets. Moreover, a group of analyzed strains reported markers related to aminoglycoside resistance (*aph, AAC, aad, ANT, armA*), sulfonamide resistance (*sul1, sul2*), trimethoprim resistance (*dfrA12* and *dfrA14*), macrolides resistance (*mphA* and *mphE*), and phenicoles resistance (*catB3* and *catL*).

### 3.4 Virulome analysis

**Figure 3:**
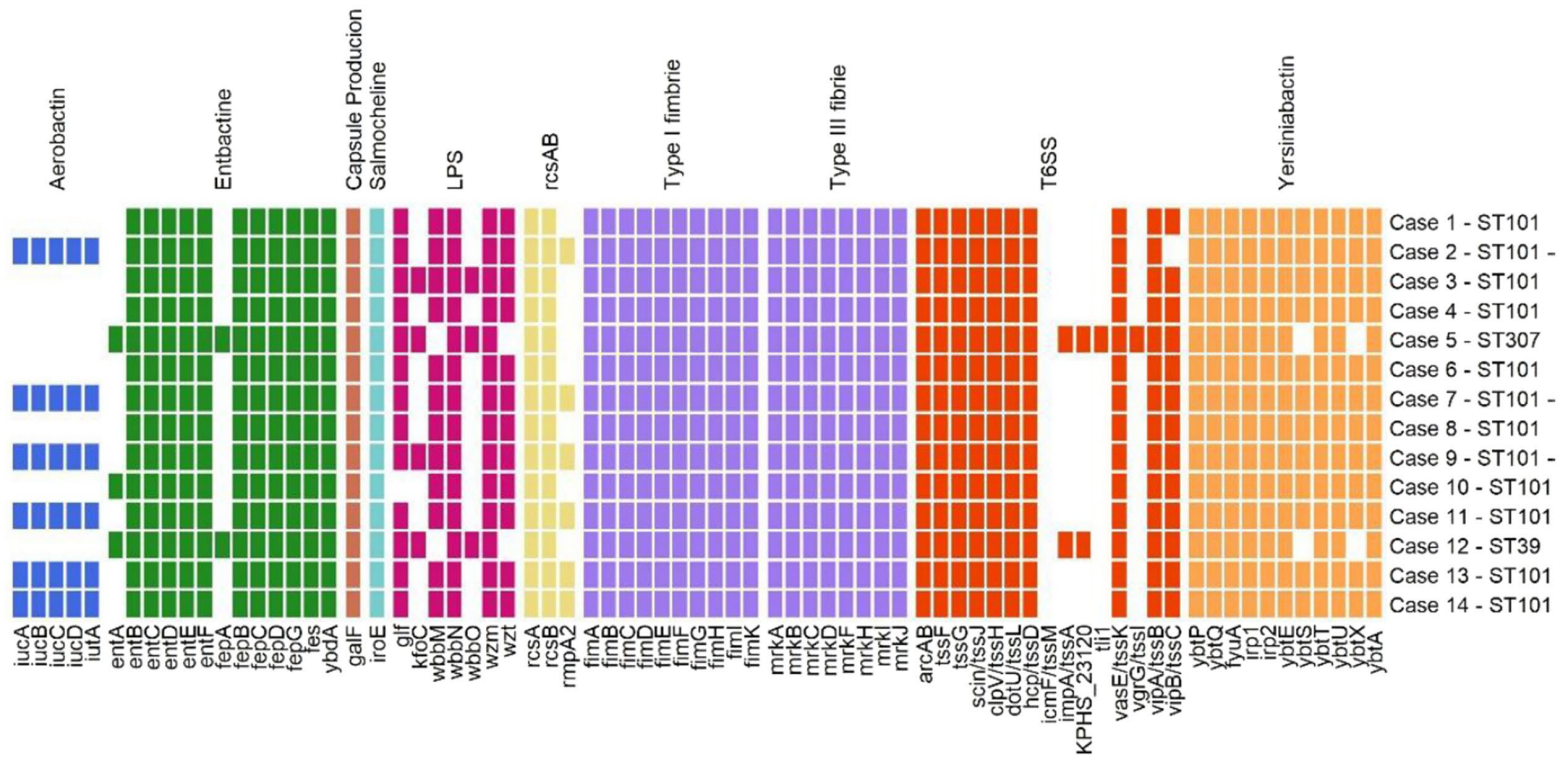
Virulome details for all the analysed *Klebsiella pneumoniae* isolates. Distribution of virulence genes across 14 different Cp-kp cases, with the genes grouped into distinct virulence categories, as indicated at the top. Each coloured square represents the presence of a specific virulence gene in a case. The categories include: Aerobactin (blue), Enterobactin (green), Capsule production (pink), Salmochelin (light blue), LPS (reddish purple), *rcsAB* (yellow), Fimbriae (type I–III) (purple), T6SS (orange), Yersiniabactin (light orange). The cases are listed on the right, with a colourful square indicating the presence of the corresponding virulence gene.

Virulence gene detection was derived from a virulence factor database (VFD), revealing an extended panel for all the analyzed isolates. Figure 2 summarizes the recorded virulence markers. All strains showed salmochelin siderophore iroE genes, capsule-production-related gene galF, *rcsA*-B system, complete gene sets for type I (fimA-B-C-D-E-F-G-H-I-K) and type III (mrkA-B-C-D-F-H-I-J) fimbriae, and type VI secretion system genes (*arcAB, tssF-G, sciN/tssJ, clpV/tssH, dotU/tssL*, *hcp/tssD, vasE/tssK*, and *vipA/tssB*). Six isolates (42.8%) documented aerobactin genes (iucA-B-C-D and iutA) and the capsule-production-related gene *rpmA2* (isolates 2, 7, 9, 11, 13, and 14). These isolates reported a negative string test result. Regarding enterobactin genes, all collected strains carried *entB*-C-D-E-F, *fepB*-C-D-G, *fes,* and *ybdA* loci. Furthermore, three isolates (21.4%) reported *entA*, and two isolates (14.3%) reported *fepA*. Focusing on yersiniabactin genes, 13 strains (92.8%) presented *ybtP*-Q-E-T-U-A genes, while 11 strains (78.6%) reported *ybtS* and *ybtX* genes. Lipopolysaccharide (LPS) synthesis genes were variably distributed among the analyzed strains. First, all the strains reported the *wbbN* and *wzm* genes, while most isolates (92.8%) had the *glf* gene. Twelve strains (85.7%) showed the *wbbM* gene, while four isolates (28.6%) revealed the *kfoC* gene and three (21.4%) the wbbO gene. For the VI secretion system, 13 isolates (92.8%) reported *vipB*/*tssC*, the same 2 strains (21.4%) (isolates 5 and 12) carried *impA/tssA* and KPHS_231 genes, and only 1 strain carried *vgrG/tssI* (7.14%) and *tli1* genes (case 005). Figure 3 summarizes the virulome analysis results.

### 3.5 Plasmids and phylogenetic correlation

The analysis identified multiple plasmids related to various antimicrobial resistance genes. Specifically, Col(pHAD28), Col156, Col440II, ColRNAI, incFIA(HI1), IncHI1B (pNDM-MAR), IncFIB(pQil), IncFIB (K), IncFIB(pNDM-Mar), IncR, IncFII (K), IncFIC(FII), and IncI1-I(Gamma) emerged from the protocol.

**Figure 4:**
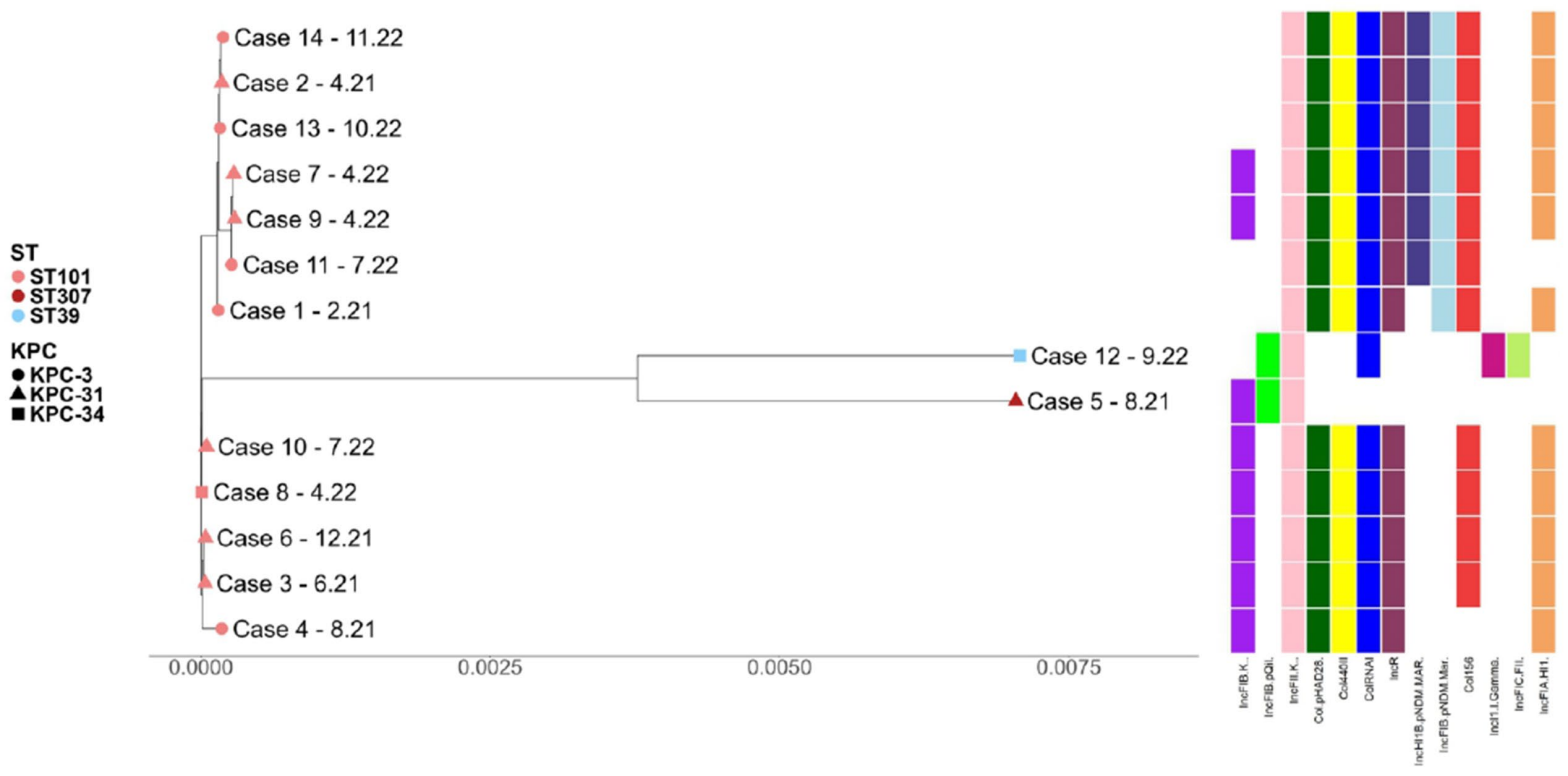
The phylogenetic tree displays the genetic relatedness among the 14 clinical isolates based on whole-genome sequencing data, revealing two major clusters. Sequence types (STs) are indicated by colored circles: pink for ST101, red for ST307, and light blue for ST39. Different *bla*_KPC_ alleles are represented by distinct shapes: circle for *bla*_KPC-3_, triangle for *bla*_KPC-31_, and square for *bla*_KPC-34_. The case identifiers are followed by the isolation date (month.year). On the right side, colored bars indicate the presence of plasmid replicons in each isolate. Each color corresponds to a specific plasmid type: yellow: Col440II, electric blue: CoIRNAI, dark green: Col(pHAD28), red: Col156, light green: IncFIB(pQil), purple: IncFIB(K), pink: IncFII(K), dark blue: IncHIIB(pNDM-MAR), light purple: IncI1-I(Gamma), magenta: IncR, light orange: InSIA(HII), light grey: IncFIB(PNDM-MAR) replicons. This figure highlights the genomic clustering of isolates and the distribution of plasmid replicons, underscoring the sequence type-specific association of mobile genetic elements potentially involved in antimicrobial resistance dissemination.

All ST101 strains carried Col(pHAD28), IncR, IncFIA(HI1), Col156, and ColRNAI; the IncHI1B(pNDM-MAR) plasmid replicons appeared in isolates 2, 7, 9, 11, 13, and 14. The ST307 showed IncFIB(K) and IncFIB (pQil) plasmid replicons. Finally, the ST39 included IncFIB (pQil), ColRNAI, IncFIC(FII), and IncI1-I(Gamma) plasmid replicons. The distribution of the different plasmid replicons. The phylogenetic tree (Figure 4) explains the relationships between different CZA-resistant isolates depending on their genetic distances. The tree branches indicate how closely related these strains are, highlighting specific features such as sequence type (ST), the presence of diverse blaKPC genes, and plasmid replicons.

According to the performed analysis, the strains could be divided into two major clusters, depending on their genetic relatedness. On the left, the image shows mostly ST101 isolates, along with sub-clusters indicating distinct groups within the same sequence types. On the right, the figure reports different sequence types (ST307 and ST39), highlighting their genetic divergence from the ST101 group. Plasmids were reported in the phylogenetic tree to support the difference between the two ST101 clusters.

## 4 Discussion

*K. pneumoniae* infections represent a significant healthcare challenge due to frequent resistance and virulence episodes. Published studies unequivocally demonstrated that clinical isolates frequently harbour both resistance and virulence genes (Muraya et al., 2022; Dong et al., 2022). These genes often correlate with specific STs, which have been previously documented among Southern Italian regions. Specifically, our study revealed a significant prevalence of ST101 according to other scientific data from the same geographical area (Loconsole et al., 2020; Mammina et al., 2012). Ceftazidime/avibactam resistance presents severe complications, drastically limiting therapeutic options (Alsenani et al., 2022). Our investigations explicitly identified distinct virulome characteristics in ceftazidime/avibactam-resistant *K. pneumoniae strains*, indicating a clear potential for hypervirulent and hypermucoviscous phenotypes (Dong et al., 2022).

We are undertaking a comprehensive analysis of these resistant strains to correlate the resistome and virulome information effectively. By using whole-genome sequencing (WGS), we reported interesting insights into our isolate collection, along with critical information regarding mobile genetic elements. Our results significantly enhanced our understanding of resistance mechanisms and the phenotypic traits of the strains. Notably, data from associated patients reveal that 50% had prior exposure to ceftazidime/avibactam, reinforcing previous findings (Aktaş et al., 2012). Otherwise, the remaining patients reported no exposure, indicating multiple pathways leading to ceftazidime/avibactam resistance. Variants of the KPC gene, particularly KPC-31, are definitively linked to ceftazidime/avibactam resistance (Guo et al., 2024), and our isolates containing KPC-31 displayed susceptibility to meropenem, consistent with findings in other ceftazidime/avibactam-resistant strains (Antonelli et al., 2019; Oliva et al., 2023). Antinori et al. (2020) described a KPC variant carrying D179Y deletion, demonstrating its ceftazidime/avibactam resistance. Our analysis registered a *K. pneumoniae* strain with the same characteristics. Moreover, KPC-31 variants occasionally evade phenotypic detection methods (Antonelli et al., 2019), highlighting the importance of integrating molecular technologies into microbiological surveillance and diagnostic workflows (Muresu et al., 2022).

Additionally, we recorded a strain with a two-amino acid deletion in the KPC-3 enzyme *Ω*-loop (positions 167–168). The above-mentioned deletion determines a mutant variant (Winkler et al., 2015) related to ceftazidime/avibactam resistance. This information leads us to hypothesize the introduction of specific phenotypic detection methods, such as chromogenic media, which previously allowed CZA resistance identification, along with targeted PCR for Ω-loop variants (Bianco et al., 2022). Livermore et al. reported ceftazidime/avibactam vulnerabilities in selecting mutations leading to resistance (Livermore et al., 2015). Furthermore, Winkler et al. documented the activity of CZA against a set of isogenic *Escherichia coli* strains carrying mutations within the U-loop of the KPC enzyme, demonstrating that substitution of amino acid at positions 165–179 enhanced ceftazidime affinity and prevented avibactam binding (Winkler et al., 2015).

During the experimental evaluations, we demonstrated some differences in KPC variants within the Italian country. Literature data reported KPC-31 also in other extended hospital settings located in Naples, Sassari, and Rome, along with KPC-66 to KPC-70, KPC-39, and KPC-49, which have not been described in Sicily (Carattoli et al., 2021; Muresu et al., 2022).

Our study evaluated membrane permeability mutations, correlating them to ceftazidime/avibactam phenotypic susceptibility profiles. Despite the presence of *ompK36* and *ompK37* mutated genes in all isolates, only some isolates registered high CZA MIC values due to a significant number of *ompK* gene mutations. Specifically, case 8 (ST101) reported numerous *ompK36* mutations along with a CZA MIC value higher than 256 mg/L. Otherwise, case 12 (ST39) revealed a mutated *lamB* protein gene along with two *ompK36* mutations, showing a ceftazidime/avibactam MIC value of 48 mg/L.

Certainly, plasmids play a fundamental role in KPC gene dissemination, along with spontaneous recombination and mutation episodes. For instance, the Tn4401 transpositions contribute to the *bla_KPC_* gene copy number increase, mobilizing them onto higher-copy-number plasmids (Cuzon et al., 2011). Our study demonstrated sequence-typing-related plasmid distribution. Among our ST101 strains, we identified the IncFIB(pNDM-Mar) and ColRNAI plasmid replicons, which are well-known in *K. pneumoniae* for carrying carbapenem-resistance genes and virulence markers such as aerobactin or *rmpA2* (Zhao et al., 2022; Henson et al., 2017; Maugeri et al., 2025). Additionally, the ColRNAI plasmid replicon was also found in ST39, demonstrating similar characteristics. We further documented the IncHI1B (pNDM-MAR) replicon, potentially associated with OXA and CTX-M resistance genes, as well as aerobactin virulence genes. Other plasmid replicons identified, including Col156 and Col440II, currently lack sufficient empirical data regarding their resistance or virulence genes.

Unique to this study, we observed the IncFIA (HI1) and ColpHAD28 plasmid replicons, suggesting strong correlations with conjugation and resistance markers (Sherburne et al., 2000). Nonetheless, connections between these replicons and known resistance or virulence elements could not be established at this stage. The IncR plasmid replicon documented in our ST101 isolates may associate with markers for aerobactin and yersiniabactin, as well as SHV resistance genes (Loconsole et al., 2024). The IncFIB(pQil) derived from ST39 and ST307, highlighting these strains’ capability to express ceftazidime/avibactam high-level resistance due to the *bla_KPC-31_* gene plasmidic localization (Loconsole et al., 2024). All our analyzed strains reported the IncFIIK plasmid replicon, which can be related to extended beta-lactamases (ESBL) production (Arcari et al., 2023). As a confirmation, all our strains revealed at least one ESBL resistance gene. All the investigations on the plasmid replicons had only an epidemiological impact in collecting preliminary information about the Inc. groups. A study limitation is the absence of a complete plasmid sequencing analysis; thus, future third-generation sequencing methodologies may be essential to define long-read analysis of plasmids. Our virulome analysis detected the *rpmA2* gene for several ST101 strains.

Remarkably, this gene correlates with capsule hyperproduction and strain hypermucoviscosity (Arena et al., 2022; Russo and Marr, 2019; Gali et al., 2023) and was not documented among *K. pneumoniae* isolates in Northern Italy. Those isolates frequently showed yersiniabactin genes, while aerobactin genes were rarely associated with clinical isolates (Thorpe et al., 2022). Our ST101 isolates also reported aerobactin and yersiniabactin genes, accounting for a Kleborate virulence score of 4. Although the negative string test result, this score may define a hypervirulent strain according to previously published data and Kleborate assessments (Arcari et al., 2023; Arena et al., 2022; Russo and Marr, 2019; Bi et al., 2018; Russo et al., 2024; Choby et al., 2020). The resistome, virulome, and plasmid analyses revealed *K. pneumoniae* strains simultaneously harbouring resistance mechanisms, virulence genes, and markers diffusion through mobile elements. As a demonstration, all the investigated STs concurrently carried beta-lactam resistance markers and at least one siderophore gene category. A consistent percentage of similar markers could be carried by a plasmid replicon, whose content may be further investigated through ultimate generation sequencing analysis.

Unfortunately, the absence of long-read sequencing methodologies was one of the study limitations, suggesting the importance of searching for external collaboration in completing experimental analysis. Furthermore, the study included a few isolates, highlighting the need to extend experimental evaluations to other patient categories.

Unfortunately, the use of short-read sequencing methodologies prevented the complete reconstruction of plasmid structures and the unambiguous assignment of virulence and resistance genes to specific plasmid backbones. This limitation underscores the importance of establishing further corroborations to implement long-read or hybrid sequencing approaches in future studies, which would allow a more accurate resolution of plasmid architecture and gene linkages. Furthermore, the study included a few isolates, highlighting the need to extend experimental evaluations to other patient categories.

A future perspective may consist of multicenter evaluations of fragile patients carrying carbapenem-resistant *K. pneumoniae* from surveillance or significant clinical samples. This perspective could emphasize the essential role of molecular surveillance protocols in characterizing and identifying particular *K. pneumoniae* clones in critical hospital settings.

## Data Availability

The original contributions presented in the study are publicly available. This data can be found here: NCBI BioProject, accession PRJNA1192262.
