# Supplementary material for: Genomic characterization of ceftazidime/avibactam-resistant Klebsiella pneumoniae: a retrospective analysis on clinical isolates from an Italian transplant unit
Source: Front Microbiol. 2026 Apr 2;17:1694693. doi: 10.3389/fmicb.2026.1694693 (PMC13082971; doi:10.3389/fmicb.2026.1694693)
Supplement: Supplementary file 1 [file Data_Sheet_1.PDF]

Isolate	Time admission to infection (days)	KPC colonization at admission	KPC acquisition from other hospitals	Prior exposure to ceftazidime/avibactam (within 60 days before the infection onset)	Type of infection	Septic shock	WARD	SOT recipient	Sample	Ascribable death
1	28	No	No	No	Pneumonia	No	ICU	Yes (liver)	Deep respiratory	No
2	125	No	No	No	Pneumonia	No	ICU	Yes (heart)	Deep respiratory	No
3	78	No	No	Yes	Intrabdominal infection with secondary BSI	No	ICU	No	Blood	Yes
4	43	Yes	Yes	Yes	Intrabdominal infection with secondary BSI	Yes	ICU	No	Blood	Yes
5	10	Yes	Yes	Yes	Pneumonia	No	ICU	No	Deep respiratory	No
6	69	No	No	No	Pneumoniae	No	ICU	No	Deep respiratory	No
7	29	No	No	No	Septic shock	Yes	ICU	Yes (kidney)	Blood	Yes
8	71	No	No	Yes	Pneumonia	Yes	ICU	Yes (liver + kidney)	Deep respiratory	Yes
9	18	No	No	No	Primary bacteremia	Yes	ICU	No	Blood	Yes
10	7	Yes	Yes	No	Pneumonia	No	ICU	No	Deep respiratory	No
11	146	No	No	Yes	Primary bacteremia	No	Cardiac surgery	No	Blood	No
12	41	Yes	Yes	Yes	Intrabdominal infection with secondary BSI	No	ICU	Yes (liver)	Blood	No
13	7	No	No	No	Urinary tract infection	No	Nephrology	Yes (kidney)	Urine	No
14	10	No	No	Yes	Urinary tract infection	No	Nephrology	Yes (kidney)	Urine	No

Supplementary Table 1. Summary of patients' clinical features and microbiological reports. Despite their recovery within a highly-specialized transplant hospital, patients underwent intensive care unit, surgery, or nephrology stays depending on their clinical conditions before or after the transplant. SOT: Solid-organ transplant; CVC: Central-venous catheter; ICU: Intensive Care Unit.

Isolate	Patient's outcome	MEM MIC (mg/ml)	CZA MIC (mg/L)	ST	*omp* alterations	KPC variant	*String test result*	*rmpA*	*ybt*	*ent*	*iro*	*iuc*	*Virulence score*	*Resistance score*
1	Survival	2	6	ST101	*ompK36-ompK37*	KPC-3	Negative	No	Yes	Yes	Yes	No	1	1
2	Death due to other reasons	2	256	ST101	*ompK36-ompK37*	KPC-31	Negative	Yes	Yes	Yes	Yes	Yes	4	2
3	Death due to infection	1	256	ST101	*ompK36-ompK37*	KPC-31	Negative	No	Yes	Yes	Yes	No	1	2
4	Death due to infection	2	48	ST101	*ompK36-ompK37*	KPC-3	Negative	No	Yes	Yes	Yes	No	1	2
5	Survival	0.13	64	ST307	*ompK36-ompK37*	KPC-31	Negative	No	Yes	Yes	Yes	No	1	2
6	Survival	2	64	ST101	*ompK36-ompK37*	KPC-31	Negative	No	Yes	Yes	Yes	No	1	2
7	Death due to infection	12	256	ST101	*ompK36-ompK37*	KPC-31	Negative	Yes	Yes	Yes	Yes	Yes	4	2
8	Death due to infection	12	256	ST101	*ompK36-ompK37*	KPC-34	Negative	No	Yes	Yes	Yes	No	1	2
9	Death due to infection	2	256	ST101	*ompK36-ompK37*	KPC-31	Negative	Yes	Yes	Yes	Yes	Yes	4	2
10	Survival	2	256	ST101	*ompK36-ompK37*	KPC-31	Negative	No	Yes	Yes	Yes	No	1	2
11	Death to other reasons	2	64	ST101	*ompK36-ompK37*	KPC-3	Negative	Yes	Yes	Yes	Yes	Yes	4	1
12	Survival	0.5	48	ST39	*ompK36-ompK37*	KPC-34	Negative	No	Yes	Yes	Yes	No	1	2
13	Survival	256	24	ST101	*ompK36-ompK37*	KPC-3	Negative	Yes	Yes	Yes	Yes	Yes	4	2
14	Survival	256	24	ST101	*ompK36-ompK37*	KPC-3	Negative	Yes	Yes	Yes	Yes	Yes	4	1

Supplementary Table 2. Summary of patients' clinical outcome and corresponding *K. pneumoniae* isolates molecular features. Specifically, *omp* indicates outer membrane proteins and *rmpA* a hypermucoviscosity-related gene. Additionally, *ybt* genes are related to yersiniabactins, *ent* genes to enterobactins, *iro* genes to salmochelins, and *iuc* genes to aerobactins. Interestingly, isolates 2,7,9,11,13, and 14 showed both *rmpA* and *iuc* genes. Abbreviations: MEM= meropenem; CZA= ceftazidime/avibactam; MIC= minimum inhibitory concentration; ST= Sequence typing. **Virulence score**: This parameter was calculated through Kleborate, taking klebsiella_abst, klebsiella_cbst and klebsiella_ybst as prerequisite and calculating a virulence score, which ranges from 0 to 5 as outlined below. Note neither the salmochelin (iro) locus nor rmpADC are explicitly considered in the virulence score, for simplicity. The iro and rmpADC loci typically appear alongside the aerobatin (luc) locus on the Kp virulence plasmids, and so presence of iuc (score of 3-5) generally implies presence of iro and rmpADC. However we prioritise iuc in the calculation of the score, as aerobactin is specifically associated with growth in blood and is a stronger predictor of the hypervirulence phenotype. The iro and rmpADC loci are also occasionally present with ybt, in the ICEKp variant - ICEKp1, but this will still score 1.Negative for all of yersiniabactin (ybt), colibactin (clb), aerobactin (iuc): virulence score 0. Yersiniabactin only: virulence score 1. Yersiniabactin and colibactin (or colibactin only): virulence score 2. aerobatin (without yersiniabactin or colibactin): virulence score 3. Aerobatin with yersiniabactin (without colibactin): virulence score 4. Yersiniabactin, colibactin and aerobactin: virulence score 5. **Resistance score**: This parameter was calculated through Kleborate calculating a resistance score, which ranges from 0 to 3 as follows: no ESBL, no carbapenemase (regardless of colistin resistance): resistance score 0. ESBL, no carbapenemase (regardless of colistin resistance): resistance score 1. Carbapenemase without colistin resistance (regardless of ESBL genes or OmpK mutations): resistance score 2. Carbapenemase with colistin resistance (regardless of ESBL genes or OmpK mutations): resistance score 3.

sample	contigs	largest contig	total length	gc (%)	n50	n75	l50	l75	n's per 100 kbp
Isolate 1	93.00	327260.00	5600550.00	56.89	183310.00	100440.00	12.00	21.00	0.00
Isolate 2	113.00	353242.00	5726413.00	56.66	142304.00	81021.00	14.00	26.00	0.00
Isolate 3	102.00	370219.00	5603131.00	57.03	142305.00	86432.00	13.00	25.00	0.00
Isolate 4	131.00	414911.00	5705099.00	56.97	139344.00	72644.00	13.00	27.00	0.00
Isolate 5	95.00	414863.00	5517958.00	57.25	145966.00	94322.00	11.00	23.00	0.00
Isolate 6	101.00	370219.00	5605127.00	57.04	142983.00	86428.00	13.00	24.00	0.00
Isolate 7	126.00	305169.00	5867025.00	56.56	129355.00	74960.00	15.00	30.00	0.00
Isolate 8	119.00	370219.00	5609988.00	57.02	139343.00	86432.00	15.00	27.00	0.00
Isolate 9	119.00	343839.00	5884239.00	56.52	142305.00	78261.00	13.00	27.00	0.00
Isolate 10	82.00	380131.00	5533499.00	57.11	197871.00	99532.00	11.00	20.00	0.00
Isolate 11	97.00	338421.00	5726012.00	56.67	143837.00	99532.00	12.00	23.00	0.00
Isolate 12	135.00	322254.00	5664051.00	57.13	107430.00	60875.00	16.00	33.00	0.00
Isolate 13	112.00	342640.00	5715433.00	56.66	141208.00	83330.00	15.00	28.00	0.00
Isolate 14	119.00	305161.00	5715502.00	56.66	139344.00	81097.00	14.00	28.00	0.00

Supplementary Table 3: quality metrics for the 14 bacterial isolates. For each sample, the table reports the total number of contigs, the size of the largest contig, the overall assembly length, GC content, N50 and N75 values, L50 and L75 indices, and the number of ambiguous bases per 100 kbp.

Sample	Average coverage depth[a]	Coverage >= 1x (%)[b]
Isolate 1	24	100
Isolate 2	26	100
Isolate 3	44	100
Isolate 4	33	100
Isolate 5	48	100
Isolate 6	46	100
Isolate 7	46	100
Isolate 8	49	100
Isolate 9	63	100
Isolate 10	67	100
Isolate 11	52	100
Isolate 12	49	100
Isolate 13	43	100
Isolate 14	30	100

Supplementary Table 4: Coverage details gathered through the QUAST (Quality assessment tool) bioinformatic platform. [a] the average depth of coverage, which is the average number of times each genome base is sequenced for genetic variations' detection and high-quality genome assemblies; [b] total number of bases with at least 1x coverage, divided by the total length assembly.
